# Intralymphatic Immunotherapy (ILIT) With Bee Venom Allergens: A Clinical Proof-of-Concept Study and the Very First ILIT in Humans

**DOI:** 10.3389/falgy.2022.832010

**Published:** 2022-03-16

**Authors:** Alexandra Chabot, Gabriela Senti, Iris Erdmann, Bettina M. Prinz, Brunello Wüthrich, Lara Šošić, Thomas M. Kündig, Pål Johansen

**Affiliations:** ^1^Department of Dermatology, University of Zurich, Zurich, Switzerland; ^2^Department of Dermatology, University Hospital, Zurich, Switzerland

**Keywords:** immunotherapy, hymenoptera, bee venom, intralymphatic administration, clinical trial

## Abstract

**Background:**

Subcutaneous venom immunotherapy (VIT) represents an effective treatment against bee venom allergy. However, it involves long treatment times, high costs, and the risk of adverse events (AEs). Shorter, safer, and cheaper treatment options are therefore pursued.

**Objective:**

To determine the safety, immunogenicity, and efficacy of bee venom intralymphatic immunotherapy (ILIT).

**Methods:**

In an open pilot study, 12 patients received bee venom ILIT in three sessions with 14-day intervals: 0.1–5 μg/dose. Ultrasound imaging was applied to guide an injection and to document the lymph node structure. In a second study, 67 patients from 15 centers in Europe and Australia were randomized to receive four doses of either 10- or 20-μg bee venom ILIT with 28-day intervals. Clinical endpoints included specific IgE and IgG and protection after a bee sting challenge. These studies were performed in the years 2000–2003.

**Results:**

In a proof-of-concept study, no serious AEs were observed. An increase in allergen-specific IgG1 but no IgG4 and IgE was observed. ILIT induced the protection against a bee sting challenge in 7 out of 8 challenged patients. In a multicenter study, an increase in allergen-specific IgG and IgE was observed, with the highest increase in patients receiving a higher ILIT dose. The study was terminated due to several serious AEs upon the sting challenge provocation after the completion of treatment. However, out of 45 patients challenged, 15 (65%) and 18 (82%) patients in the 10- and 20-μg group, respectively, showed an improvement of two grades or more. No correlation was observed between antibody levels and sting protection.

**Conclusions:**

While a pilot study suggested the safety and efficacy of bee venom ILIT, a high number of AEs seen after the sting challenge following a randomized study indicate that the immunology protection offered by bee venom ILIT is insufficient. Of note, the bee venom allergen extract used in the two studies were from the two different providers. While the first study used a formulation approved for use in subcutaneous VIT, the second study used a nonapproved formulation never tested in humans. Further studies on approved formulations should be performed to generate conclusive results regarding the safety and efficacy of bee venom ILIT.

## Introduction

Bee stings can induce systemic allergic and potentially life-threating reactions in sensitized individuals. In Europe, insect stings cause 48.2% of all anaphylactic reactions in adults (1). While emergency medication consisting of epinephrine, antihistamine, and cortisone is of uttermost importance in the personal disease management, subcutaneous venom immunotherapy (VIT) is an effective and the only disease-modifying treatment. VIT can protect sensitized patients from subsequent systemic reactions, prevents morbidity, and improves health-related quality of life (2). However, VIT is frequently associated with allergic side effects, long treatment times, and high costs, for which reason the development of novel therapeutic modalities is of great personal and clinical importance (3). Moreover, although the compliance to VIT is typically higher for the treatment of allergies to pollen, animal dander or dust mite, more than 50% of the discontinuations are caused due to the lack of compliance and inconvenience (4).

The aim of any allergen immunotherapy (AIT), including VIT, is to deliver allergens to the lymph nodes for the induction of anti-allergen immune responses in B- and T-cells. We and others have shown that only a minor fraction of the AIT material injected subcutaneously reaches the draining lymph nodes (5–7). Hence, a direct injection of the AIT material into a subcutaneous lymph node with its higher density of antigen-presenting cells (APCs) as well as T- and B-cells should facilitate stronger immune responses against AIT allergens. Because of the more efficient delivery to the lymph node, this so-called intralymphatic immunotherapy (ILIT) also has the potential of lowering the doses of allergen required to induce protective B- and T-cell responses. Furthermore, because the allergen is better contained in the lymph node than in the subcutaneous tissue and because mast cells and basophils are almost absent in lymph nodes, ILIT is less likely to trigger allergic mast cell reactions than subcutaneous AIT and hence potentially less adverse events (AEs) (8–10). Therefore, we assume that ILIT requires fewer injections and a reduced treatment duration with comparable efficacy to conventional VIT. This may generate cost savings and is of greater convenience to the subject due to a lower number of treatments over a shorter timeframe.

Intralymphatic immunotherapy has been tested in clinical phase I and II studies in patients with sensitization to grass and tree allergens (7, 8, 11–14). Here, pollen allergen ILIT has shown a clinical improvement after only three injections within a timeframe of 4–12 weeks. By comparison, the conventional subcutaneous immunotherapy (SCIT) typically requires 20–50 injections over 3–4 years to induce the targeted effect. In children and adults with bee venom sensitization and systemic bee sting reactions, bee venom VIT is highly recommended, but long-term effectiveness typically requires 5 years of treatment (15–18). According to an expert consensus, a total of approximately 100 injections are usually given every 4 weeks in the 1st year of treatment, every 6 weeks in the 2nd year, and every 8 weeks from years 3 to 5 and for the following years when lifelong VIT is necessary (19). So far, no human clinical trials on bee venom ILIT have been published. However, in murine experiments, bee venom ILIT enhanced allergen-specific B- and T-cell responses when compared to conventional subcutaneous administration (5).

Therefore, and as a proof of principle of venom ILIT in humans, we performed two studies. In a pilot study, 12 patients with bee venom allergy received bee venom ILIT and were monitored for safety, immunogenicity, and treatment efficacy. In a following randomized study, 67 patients were split into two dose arms.

The two studies were performed almost 20 years ago and were the very first ILIT trials in humans. Hence, the trials also represent a historical witness and thereby represent a milestone in the historical development of AIT in humans. Originally, we did not submit the data for publication, the major reason being the many events of anaphylactic reactions in a larger randomized study. However, we are frequently asked to share the data with colleagues and at scientific and clinical meetings. Moreover, after several phase I/II clinical trials with grass pollen allergen ILIT, the first phase III trial is currently running for the purpose of getting general approval for the use of ILIT in treating hay fever (EURDACT: 2020-001060-28). Hence, we now consider it as important and timely appropriate to share the results, observations, and the experiences of these first-in-human ILIT trials with scientists and allergologists: ILIT in general, but also bee venom ILIT work as an alternative delivery method to conventional and laborious VIT for the treatment of bee venom allergy.

## Methods

### Study Design and Patient Characteristics

To characterize the safety and efficacy of ILIT in patients with a history of systemic allergic reactions to honeybee stings, two clinical studies were performed.

**Figure 1:**
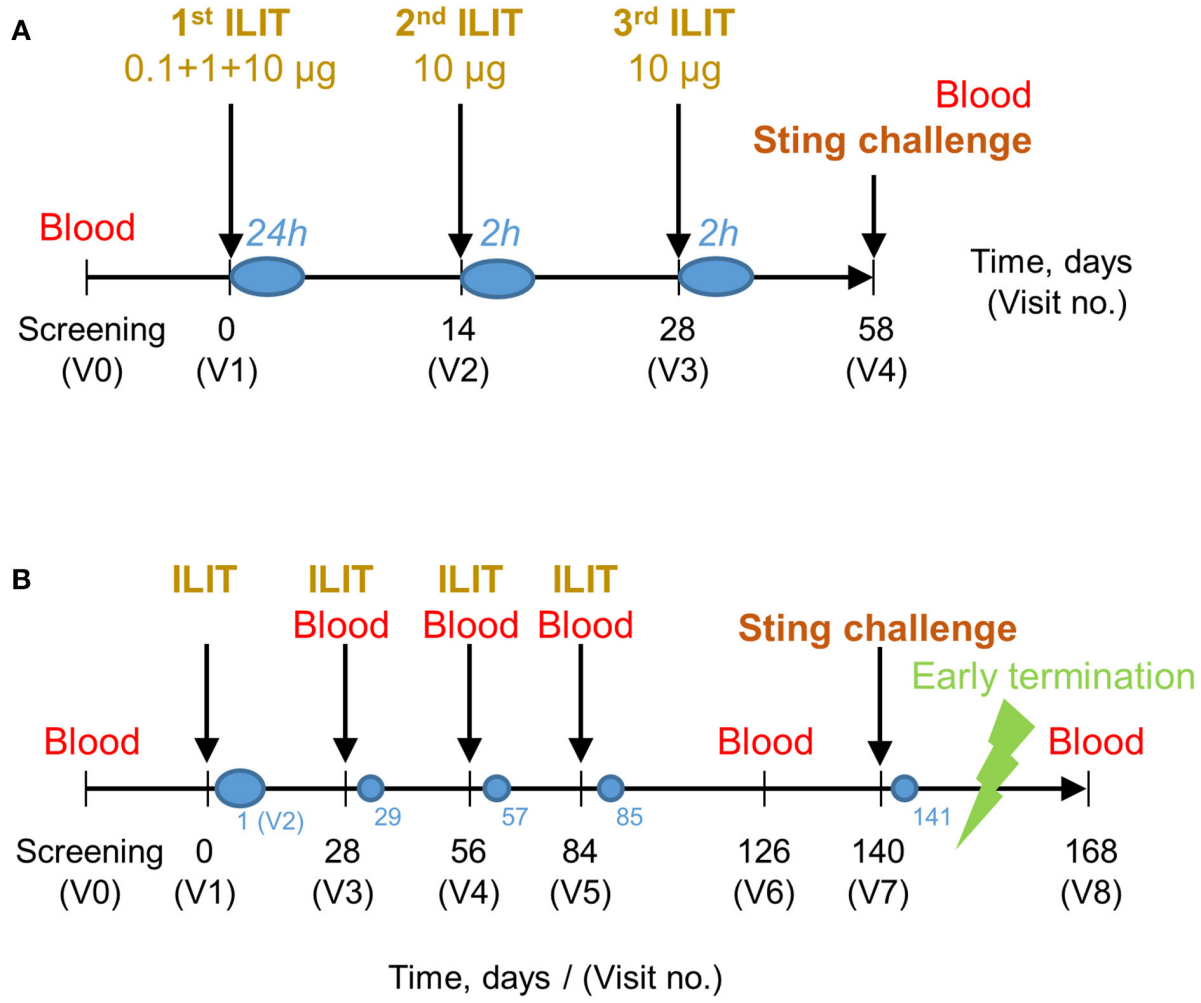
Time scheme of pilot **(A)** and randomized **(B)** bee venom intralymphatic immunotherapy (ILIT) studies. **(A)** After screening (V0), patients in the pilot study attended a first visit (V1, day 0) where they received the first injections of aqueous bee venom in doses of 0.1, 1, and 5 μg with 30-min intervals, followed by 5 μg of aluminum hydroxide-absorbed bee venom. During the second (V2) and third (V3) injection visit, 5 μg of aluminum hydroxide-absorbed bee venom was injected with 30-min intervals. The total cumulative dose was 31.1 μg. After each visit, patients were observed at the intensive care unit or the normal ward for 24 or 2 h (blue circles). At the screening visit (V0) and on day 58 (V4), venous blood was drawn and a honeybee sting challenge was performed. **(B)** Following the screening visit (V0) of the randomized study, patients in the randomized study attended a first visit (V1, day 0) where they received bee venom ILIT injections: 0.1, 1, and 10 μg 30 min apart, provided no systemic signs or symptoms. Visit 2 was a safety follow-up 1 day after visit 1 (blue circle). At visit 3, treatment group 1 received one 10-μg dose and treatment group 2 received two doses of 10 μg 30 min apart. At visits 4 and 5, one treatment group received 10 μg and the other group received 20 μg in a single dose. The total cumulative doses were 41.1 and 71.1 μg. Blood was drawn at screening and at every visit from visit 3 onward as to measure bee venom-specific immune responses. A deliberate bee sting challenge was performed at visit 7. Visits 3, 4, 5, and 7 were followed up for safety 1 day later by telephone (small blue circles).

The first ILIT study with a bee venom allergen was an open pilot study in patients with a history of allergic reactions after a bee sting (cf. scheme in Figure 1A). In total, 12 patients were enrolled after the assessment of eligibility. Written informed consent was obtained from all patients prior to enrolment. The study was performed from October 2000 to July 2001 at the University Hospital Zurich, Switzerland, approved by the independent ethics committee [IEC; (#2000–366 and #2001–422)], notified with *Swissmedic*, registered in the University Hospital Zurich trial register, and conducted according to the International Council for Harmonization's guidelines of Good Clinical Practice (ICH-GCP) and the Declaration of Helsinki.

The second study was a randomized multicenter international phase I/II dose-comparison study of bee venom allergen ILIT (cf. scheme in Figure 1B). In total, 88 patients were assessed for eligibility, after which 67 patients were randomized to either one of the two dose arms. All patients signed written informed consent prior to enrolment. The study was conducted from January 2002 to June 2003 at four centers in Australia, three centers in Austria, four centers in Germany, three centers in Spain, and one center in Switzerland. Study protocols, amendments, and all related documentations were approved by the local IECs and notified by Swissmedic and other national medical product agencies prior to study initiation.

The patient inclusion criteria for both studies were 18–55 years of age, a history of systemic allergic reaction after honeybee sting (specified by grade III or IV reactions in the first pilot study), a positive intradermal test, and positive serum IgE specific to honeybee venom. Exclusion criteria for both studies were HIV positivity, pregnancy, cutaneous or systemic mastocytosis (serum tryptase ≥ 16 μg/L), clinically significant cardiovascular or pulmonary disease, active infectious disease, clinically significant hepatic disease, renal disease, a history of malignancy, autoimmune disease, organic brain syndrome, or a significant psychiatric disorder, severe hematological abnormality, current treatment with β-blockers, angiotensin-converting-enzyme inhibitors, or immunosuppressive agents, severe asthma uncontrolled by pharmacotherapy and/or irreversible airway obstruction, and a body mass index ≥ 30 kg/m^2^. Other inclusion and exclusion criteria followed the recommended standards for VIT.

### Allergens

For a pilot study, commercially available bee venom allergen extracts were purchased from ALK-Abelló (Hamburg, Germany). A non-depot aqueous formulation (Pharmalgen®) was used for the initial ILIT session, while an aluminum hydroxide containing depot formulation (Alutard®) was used for the later ILIT sessions. For both Pharmalgen and Alutard, the maintenance preparations containing 100 μg *Apis mellifera* bee venom allergen extract were applied.

For a randomized study, an investigational product (*BeeAlleVax*™) was provided by MannKind Corp. (Westlake Village, CA, USA); of note, this product is no longer available to receive or use. The allergen extract contained naturally occurring protein and polypeptide venom from the honeybee *A. mellifera*, the major allergenic components of which are phospholipase-A_2_ (PLA_2_), hyaluronidase, and melittin. The lyophilized product was formulated with 5% sucrose, 0.01% polysorbate 80, 0.01% poloxamer 188, and 0.0005% citric acid. The vaccine was reconstituted in aluminum hydroxide adjuvant (2% Alhydrogel from Brenntag, Fredriksund, Denmark) prior to administration.

### Intralymphatic Immunotherapy

In a pilot study, a subcutaneous lymph node of the inguinal area was catheterized with a 28-gauge needle under sonographic guidance. To prevent inadvertent intravascular administration, the injections of 100-μl bee venom allergen extracts were given after aspiration. For safety reasons, the administrations took place at the intensive care unit and the first dose of bee venom allergen was fractionated in doses of 0.1, 1, and 5 μg with 30-min intervals and using a nondepot aqueous formulation (Pharmalgen®). The nondepot formulation facilitated the monitoring of any allergic side effects that would occur and vanish rapidly while the patient was still in the intensive care unit. Once the treatment tolerance had been confirmed, 5 μg of bee venom allergens adsorbed on aluminum hydroxide (Alutard®) were administered 30 min later. The injected lymph node was monitored sonographically for swelling. After 4 h, the patient was transferred to the normal ward, where general monitoring continued for at least 24 h before the patient was discharged. After 2 and 4 weeks of the first ILIT visit, patients returned to receive two further injections of 5 μg of bee venom allergens adsorbed on aluminum hydroxide, with a 30-min interval between the injections (Figure 1A). Vital signs of every patient were monitored for 2 h after each ILIT visit at the normal ward. The total dose of the injected allergens after three ILIT visits was 31.1 μg.

In the second study (cf. a scheme in Figure 1B), patients were randomized to receive either 10- or 20-μg bee venom allergen extract adsorbed to aluminum hydroxide (*BeeAlleVax*™) ILIT by injections into a superficial inguinal lymph node using sonographic guidance as described for the abovementioned pilot study. Nine visits were scheduled, including a screening visit. At visit 1, both treatment arms received 0.1-, 1-, and 10-μg bee venom allergen extract with 30-min intervals, providing no signs of systemic reactions. A safety visit followed 1 day later. After 4 weeks, of the first ILIT, one study arm received a single ILIT dose of 10-μg allergens, while the second study arm received two doses of 10 μg, 30 min apart. After 8 and 12 weeks of the first ILIT, one study arm received a single ILIT dose of 10 μg, while the second study arm received a single dose of 20 μg. Each patient was observed at the normal ward, 4 h after the first ILIT and 2 h after the next three ILIT visits. During the observation time, vital signs were recorded, flare and wheal reaction at the injection site was measured and spirometry was performed. Blood was drawn at screening and from visit no. 3 (second ILIT) onward for the analysis of IgG, IgE, and tryptase measurement, and for further investigation of hematology and chemistry panels. Urinalysis, including urine test strip, urine sediment, and pregnancy tests, were performed at screening and from visit no. 3 onward. After ILIT sessions no. 2–4 (visits 3–5), patients were followed up 1 day later by telephone. The total dose of injected allergen after the four sessions of ILIT was 41.1 μg in the 10-μg group and 71.1 μg in the 20-μg group.

### Bee Sting Challenge

To test the efficacy of bee venom allergen ILIT, study participants were offered to receive a sting provocation with a honeybee. The bee sting challenges were performed as recommended by the European Academy of Allergology and Clinical Immunology (EAACI) and the reaction graded according to the Mosbech–Muller system (20). In a pilot study, the challenge was performed 4 weeks after having completed the bee venom ILIT. In a randomized follow-up study, the challenge was performed 8 weeks after the completion of bee venom ILIT. No challenge was performed at baseline, but a patient history record of allergic reactions was applied to analyze the effect of ILIT on the protection from sting reactions.

Briefly, sting provocation was performed in an emergency setting with appropriate rescue equipment and trained personnel. The honeybee was forced onto the patient's skin using a syringe and left for 1 min on the skin surface. The bee stinger was left on the skin but removed immediately if a systemic reaction occurred. After 5, 15, and 60 min, the sting site was examined and the findings were documented. A peripheral intravenous catheter was placed prior to the sting provocation and not removed until the end of the observation period. Vital signs such as pulse, blood pressure, and oxygen saturation were continuously monitored. Anaphylactic reactions were immediately treated depending on the degree of severity and according to internal emergency guidelines of the allergy unit.

### Laboratory Investigations

In a pilot study, patient examinations were performed at an interval of ~2 weeks. Hematological and chemical laboratory tests as well as urinalysis were carried out. PLA2-specific IgG1, IgG4, and IgE, as well as tryptase in sera were analyzed by an in-house sandwich ELISA method to determine titers. In a multicenter study, honeybee venom-specific IgE (RAST, ImmunoCAP, or categorized) and IgG (SPRIA or categorized) was measured at the indicated time points.

### Primary Objectives

The primary objective of both studies was to evaluate the safety of bee venom ILIT in patients with a history of bee venom allergy. Hence, both studies had an open-label design. Particularly, this pilot study had the intention to show the tolerability of bee venom ILIT and potential AEs of this novel method for bee venom VIT in humans.

In this pilot study, patient examinations were performed on days 0, 1, 14, 15, 28, 29, and 42. Cardiovascular examinations included heart rate, blood pressure, and ECG. Furthermore, the respiratory rate and respiratory peak flow as well as skin, ear, nose, throat, neck, thyroid, cardiopulmonary function, lymph nodes, the nervous system, body weight, and body temperature were examined. AEs and severe adverse events (SAEs) were recorded at every observation after each injection.

In a randomized multicenter study, the primary objective was to determine the safety of bee venom ILIT with the specific investigational product *BeeAlleVax*™. Safety assessments consisted of monitoring and recording AEs and SAEs, the regular measurement of vital clinical signs, respiratory function, and physical examination, regular monitoring of hematology, serum chemistry, pulmonary function, renal function, and urinalysis. Allergic skin flare and wheal reactions and systemic reactions were evaluated for frequency, severity, and the time of appearance. All adverse treatment reactions that occurred from the first ILIT until 4 weeks post last ILIT were recorded. AEs were recorded in Case Report Forms and were assessed by an investigator as definitely, probably, possibly, or not related to ILIT. The treatment was discontinued when a patient suffered a grade IV allergic reaction.

Severe adverse events were defined as AEs that were potentially life-threatening, required inpatient hospitalization and the prolongation of hospitalization with systemic treatment, or resulted in a persistent or significant disability with an effect on daily activities, including immune system disorders, psychiatric disorders, vascular disorders, gastrointestinal disorders, respiratory and thoracic disorders, general disorders and injection site conditions, and skin and subcutaneous tissue disorders. The monitoring of AEs included chest constriction, dyspnea, hyperventilation, desaturation, the loss of consciousness, precordial pain, bradycardia, severe hypotension, severe nausea and vomiting, vertigo, dizziness, angioedema, dysesthesia, generalized urticaria and erythema, flush, anxiety, and fatigue.

### Secondary Objectives

The major secondary objective was to show the efficacy of bee venom ILIT. For this, a bee sting challenge with a honeybee was performed as described above. Furthermore, bee venom-specific IgG, IgE, and tryptase were determined in sera by an ImmunoCAP analysis. In a pilot study, complete blood counts were tested along with chemistry laboratory tests and urinalysis. In a multicenter study, secondary objectives were to compare vaccine doses for efficacy by the bee sting challenge, and to assess the allergen-specific IgE and IgG as surrogate endpoints for efficacy.

### Statistical Analysis

The primary goal of this pilot study was to test the safety of bee venom ILIT in humans. Because only limited information was available for a quantitative valid sample-size estimation, the study was designed as an experimental pilot phase open study. A sample size of 12 patients was estimated to be sufficient to produce valuable information to determine the patient sample size for an subsequent phase I/II study, the primary objective of which would be to further demonstrate dose-dependent safety (21). The Wilcoxon matched-pairs signed-rank test was used for a statistical analysis of antibody and sting challenge data before and after ILIT.

In the multicenter trial, study participants were randomized into two treatment arms by using a random permuted block design, which should minimize the risk of confounder effects. The multicenter design of the study also ensured that individual sites were unable to discern blocking patterns. Within-group comparisons of antibodies were calculated with the paired-samples *t*-test or the Friedman test for more than two related variables. The sting reactions were scored, and the effect of ILIT and dose were analyzed statistically using a mixed-effects model. The Wilcoxon matched-pairs signed-rank test was used for the analysis of sting challenge data before and after ILIT. A sub-analysis of antibodies in sting-protected (*n* = 35) and nonprotected (*n* = 10) patients was made using the Mann–Whitney *U* test. Due to the early termination of this study, an abbreviated clinical study report focusing mainly on safety was produced. Therefore, only summary statistics were provided to describe the results. All analyses were made using the GraphPad Prism 9.00 software.

## Results

### Patient Characteristics, Pilot Study

In a monocentric pilot study, 12 patients with bee venom allergy grade III were recruited and received complete per-protocol bee venom ILIT. Of these, eight out of 12 patients completed the bee sting challenge period 4 weeks after the last ILIT. Four patients refused of a deliberating bee sting.

### AEs, Pilot Study

**Figure 2:**
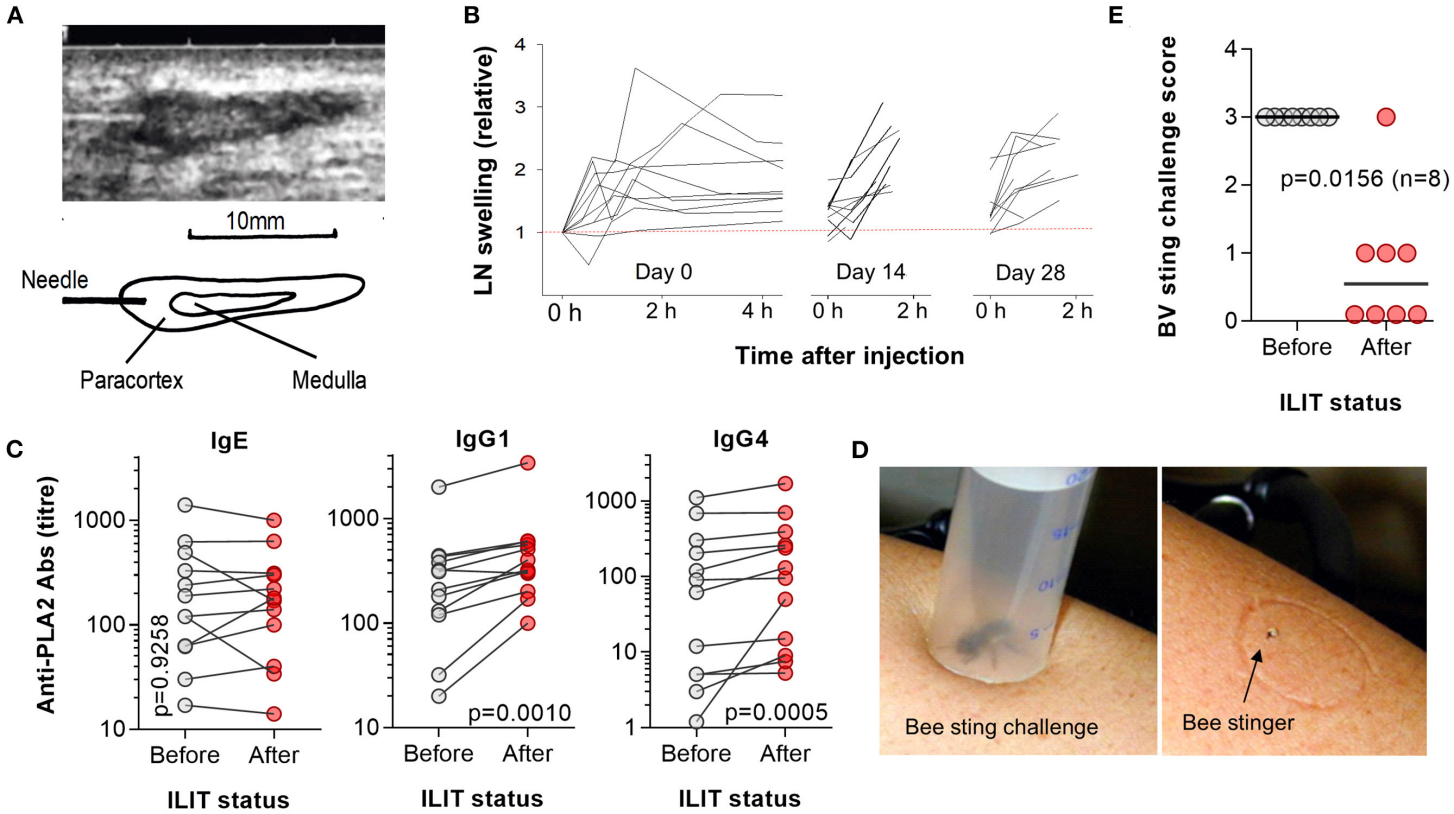
Assessment of node swelling, antibody responses, and honeybee provocation test. **(A)** Ultrasound imaging was applied to guide an injection and to document possible changes in the lymph node structure. **(B)** Sonographic monitoring of lymph node swelling for 2–4 h post first ILIT injection at each of the three ILIT sessions on days 0, 14, and 28. **(C)** Patient blood analyzed prior to the first bee venom ILIT and 4 weeks after the last ILIT. The sera were measured by ELISA for IgE, IgG1, and IgG4 antibodies against the major bee venom allergen PLA2 and expressed as titer. Statistical differences were analyzed by Wilcoxon matched-pairs signed-rank tests. **(D)** A honeybee was trapped in a prepared syringe, the plunger lowered to provoke a sting, and the stinger left on the skin for 60 min while monitoring local and systemic reactions. **(E)** Eight patients were subject to a bee sting challenge prior to the first bee venom ILIT and 4 weeks after the last ILIT. While all patients exhibited grade III allergic reactions to bee stings prior to vaccination, after treatment, only one grade III reaction and three cases of grade I reactions occurred. In the remaining four patients, there was no systemic allergic reaction to the challenge. The value of *p* was calculated by Wilcoxon matched-pairs signed-rank tests.

In a pilot study, no AEs or SAEs were observed between the first and last bee venom ILIT injection, or in a 4-week period between the last ILIT and the bee sting challenge. Moreover, there were no major changes in vital clinical signs or physical findings. Shortly after the first injection of the allergen, the treated lymph nodes significantly increased in volume as monitored from the sonographic images (Figure 2A). The swelling reached a maximum level after 1–3 h when the volume increased by 2–4-fold (Figure 2B). Of note, the degree of swelling did not return to day 0 baseline levels within a 14-day interval between the two injections; however, new swelling reactions were observed after the booster injections 2 and 4 weeks later. Upon palpation, the lymph nodes appeared to be enlarged, indurate, and slightly tender.

There were no relevant changes in hematological and chemistry laboratory tests and urinalysis (data not shown). Patients were also asked to compare the level of pain of an ILIT injection into a lymph node to the venous puncture during the same visit. Intralymphatic injections were rated as less painful than venous puncture and comparable with subcutaneous injections (8). Of note, as the sensory innervation of lymph nodes is sparse, any pain arose solely from penetrating the skin.

### Serology Analysis, Pilot Study

To test if bee venom ILIT affected the level of IgE and to test the immunogenicity of bee venom ILIT, blood collected prior to first ILIT session and 4 weeks after the last ILIT session of a pilot study were measured for PLA2-specific IgE and IgG (Figure 2C). While no significant changes in IgE titers could be determined, bee venom ILIT caused a significant increase in PLA2-specific IgG1 antibody levels (*p* = 0.0010) and IgG4 (*p* = 0.0005). After 8 weeks, the average increase in titers was 2.2-fold for IgG1 with 11 out of 12 patients showing an increase, and 4.9-fold for IgG4 with all patients showing an increase. Importantly, bee venom-specific IgE levels were not boosted.

### Bee Sting Challenge, Pilot Study

Eight patients from a monocentric pilot study were subject to a bee sting challenge 4 weeks after having completed the bee venom ILIT (Figure 2D). Bee venom ILIT improved the protection from the challenge as compared to a sting challenge at the screening session and prior to the first ILIT injection (Figure 2E; *p* = 0.0156; marginal homogeneity test). While all patients exhibited grade III allergic reactions to bee stings prior to vaccination, only one grade III reaction and three cases of grade I reactions occurred after completing bee venom ILIT. In four patients, no systemic allergic reaction to the challenge was observed.

### Patient Characteristics, Randomized Study

**Figure 3:**
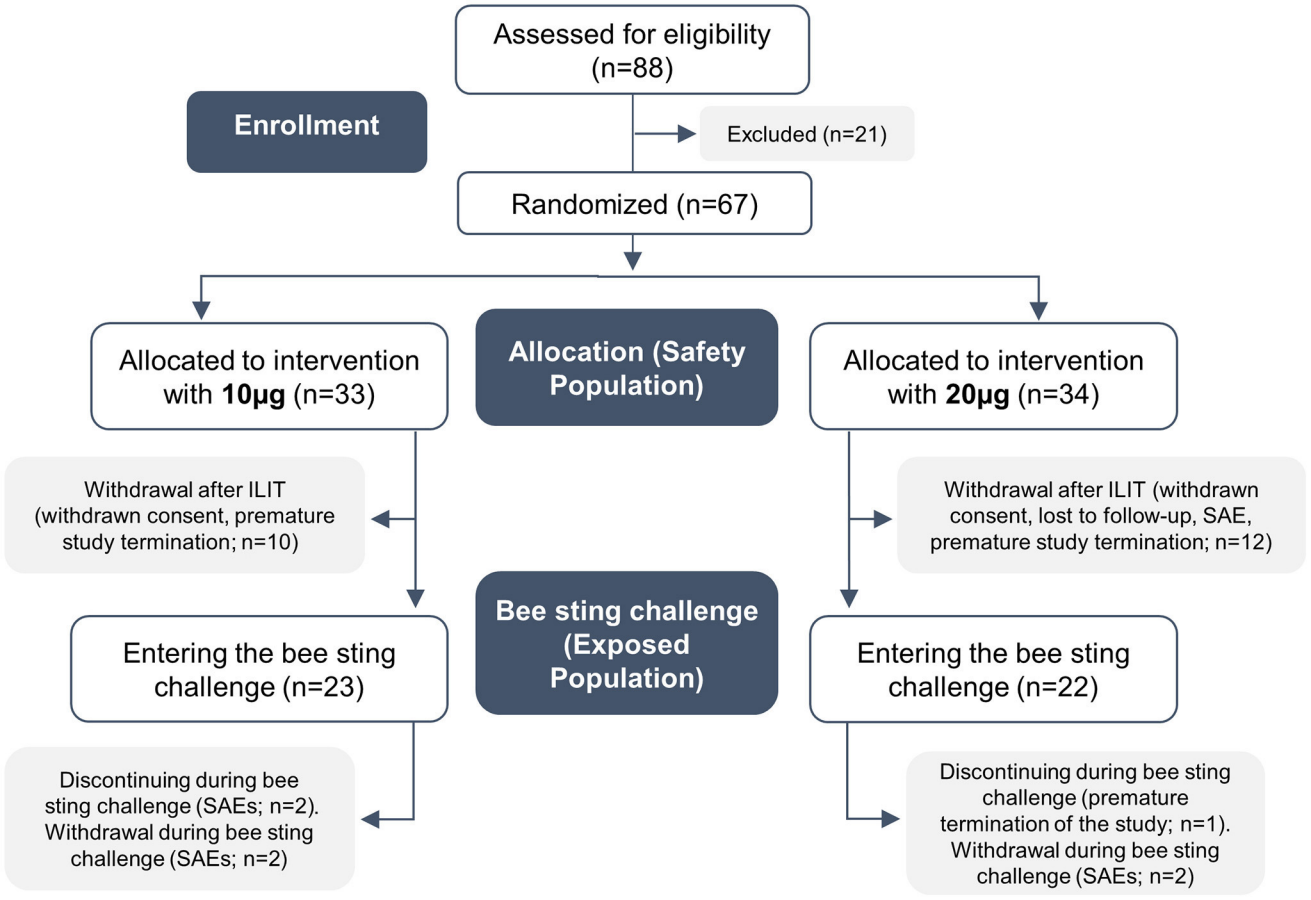
Flow chart illustrating the development of cohorts and patients in this study.

In the multicenter randomized study, 88 patients were found to be eligible after initial screening, and 67 of these were randomized to receive 10- (*n* = 33) or 20-μg (*n* = 34) bee venom allergen ILIT (cf. flow chart in Figure 3). All randomized patients were included in the safety evaluation. Gender ratio, age, and race were equally distributed in both study arms of the safety population, with a grade III allergy as the most frequent historical bee venom reaction in both treatment groups (Table 1). The time interval between the two injections doubled from 14 to 28 days as compared with a pilot trial. The reason for this was that lymph node swelling last for longer than 14 days and to comply with general recommendations in vaccine immunology (22, 23). Out of the 67 study participants that received bee venom ILIT, 45 (67%) were challenged in a deliberate honeybee sting provocation test; 23 patients in the 10-μg treatment arm and 22 patients in the 20-μg treatment group. Hence, a total of 22 patients, 10 in the 10-μg group and 12 in the 20-μg group, withdrew from attending the bee sting challenge after finishing the ILIT treatment. The primary reason for not entering the final sting provocation test was the premature termination of the study by the sponsor on April 11, 2003 after several SAEs following the sting test: seven patients out of 33 in the 10-μg treatment group and nine patients out of 34 in the 20-μg treatment group. Two patients in the 10-μg and one patient in the 20-μg withdrew consent. One patient in the 20-μg group was lost to a follow-up. One patient in the 20-μg treatment group withdrew due to a SAE of anaphylactic reaction during the provocation test. One patient underwent treatment up to visit 7, but did not receive a bee sting owing to the fact that he received 14 country stings in the period March 13, 2003 to April 9, 2003 without any adverse reactions and did not take any medication. None of the patients were lost with respect to noncompliance.

**Table 1:** Baseline characteristics and demographics for study subjects included in a randomized multicentric bee venom intralymphatic immunotherapy (ILIT) trial.

**Characteristics**	**10 μg ILIT *N* = 33**	**20 μg ILIT *N* = 34**
**Gender** ***n*** **(%)**		
Male	27 (81.8%)	22 (64.7%)
Female	6 (18.2%)	12 (35.3%)
**Age (years)**		
Mean (SD)	38.1 (10.2)	33.1 (10.7)
Median	40.0	31.5
Min-Max	18–58	18–60
**Race** ***n*** **(%)**		
Caucasian	33 (100%)	33 (97.1%)
Hispanic	0 (0.0%)	1 (2.9%)
Black	0 (0.0%)	0 (0.0%)
Asian	0 (0.0%)	0 (0.0%)
Other	0 (0.0%)	0 (0.0%)
**Systemic Allergic Reaction Grade**		
Grade 1	1 (3.0%)	0 (0.0%)
Grade 2	4 (12.2%)	5 (14.7%)
Grade 3	21 (63.6%)	25 (73.5%)
Grade 4	7 (21.2%)	4 (11.8%)

In each of the 10- and 20-μg treatment arms, 21 patients completed all planned ILIT injections as well as the bee sting challenge. Two patients in the 10-μg treatment group withdrew due to SAEs in the ILIT period: one patient suffered an anaphylactic shock after ILIT and one patient withdrew consent due to an allergic reaction after an accidental insect sting while at work. One patient in the 20-μg treatment group withdrew consent due to premature termination of the study by the sponsor.

### AEs, Randomized Study

In the randomized study, 25 out of 33 patients (75.8%) in the 10-μg treatment and 24 out of 34 patients (70.6%) in the 20-μg treatment experienced at least one AE during the treatment period (Table 2). The AEs of 18 patients in the 10-μg study arm and 19 patients in the 20-μg study arm were assessed as causally related to the study medication, e.g., to bee venom ILIT. All AEs were characterized as mild. The most affected system organ class was the general disorders and administration site conditional class, followed by skin and subcutaneous tissue disorders and infections and infestations. Injection site swelling and injection site pain were the most common preferred terms within the general disorders and administration site conditional system organ class. No statistical differences were determined when comparing the two treatment arms of 10 and 20 μg and with regard to the affected system organ class.

**Table 2:** Adverse events (AEs) in the safety population (reported for ≥5% of subjects in either treatment groups) during the treatment period by the preferred term.

**Preferred Term**	**10 μg *N* = 33**	**20 μg *N* = 34**
Number of Subjects with at least one AE	25 (75.8%)	24 (70.6%)
Pruritus NOS	7 (21.2%)	5 (14.7%)
Flushing	6 (18.2%)	2 (5.9%)
Injection site pain	4 (12.1%)	4 (11.8%)
Injection site swelling	3 (9.1%)	5 (14.7%)
Headache NOS	2 (6.1%)	4 (11.8%)
Injection site erythema	3 (9.1%)	2 (5.9%)
Nasopharyngitis	2 (6.1%)	3 (8.8%)
Urticaria drug-induced	1 (3.0%)	4 (11.8%)
Allergy to insect sting	3 (9.1%)	1 (2.9%)
Chest tightness	2 (6.1%)	2 (5.9%)
Dizziness	1 (3.0%)	2 (5.9%)
Influenza	0 (0.0%)	3 (8.8%)
Injection site edema	2 (6.1%)	0 (0.0%)
Tachycardia NOS	2 (6.1%)	0 (0.0%)
Cannula site reaction	0 (0.0%)	2 (5.9%)
Erythema	0 (0.0%)	2 (5.9%)
Injection site discomfort	0 (0.0%)	2 (5.9%)
Lymphadenopathy	0 (0.0%)	2 (5.9%)
Rhinitis allergic NOS	0 (0.0%)	2 (5.9%)
Rhinitis NOS	0 (0.0%)	2 (5.9%)

Seven patients had at least one SAE during the treatment period. Five out of seven patients with at least one SAE had an immune system disorder SAE. In the 10-μg treatment group, SAEs were allergic reaction grade II and anaphylactic allergic reaction grade III. In the 20-μg treatment group, the SAEs were grade II systemic reaction, grade III allergic reaction following ILIT, and anaphylaxis. Four treatment period SAEs were assessed by an investigator as having a definite relationship to the study drug. One subject experienced a severe systemic reaction (grade II) in response to an accidental bee sting while at work, one patient was diagnosed with prostate adenocarcinoma, and one patient had a motorbike accident during the treatment period. These SAEs were considered as not related to study medication by an investigator. None of the treatment period SAEs were attributed to the method of vaccine delivery.

Nevertheless, no safety issues were identified with regard to vital signs, respiration rate, respiratory spirometry, and ECG assessment. Changes were seen in systolic and diastolic blood pressure and heart rates prior to the bee sting challenge at day 140, which could possibly be related to subject anxiety. No major changes, patterns over time, or differences between 10- and 20-μg treatment groups were identifiable with regard to vital signs and physical findings. Erythema was most often observed in 15–30 min post-injection regardless of visit number, bee venom allergen dose, and the number of injections received during a visit. Independent of the bee venom ILIT dose, edema at the site of injection was generally observed in 30–120 min post-injection. Further interpretation of the results for erythema, edema, and the surrogate efficacy markers is difficult due to the early termination of this study.

### Serology, Randomized Study

**Figure 4:**
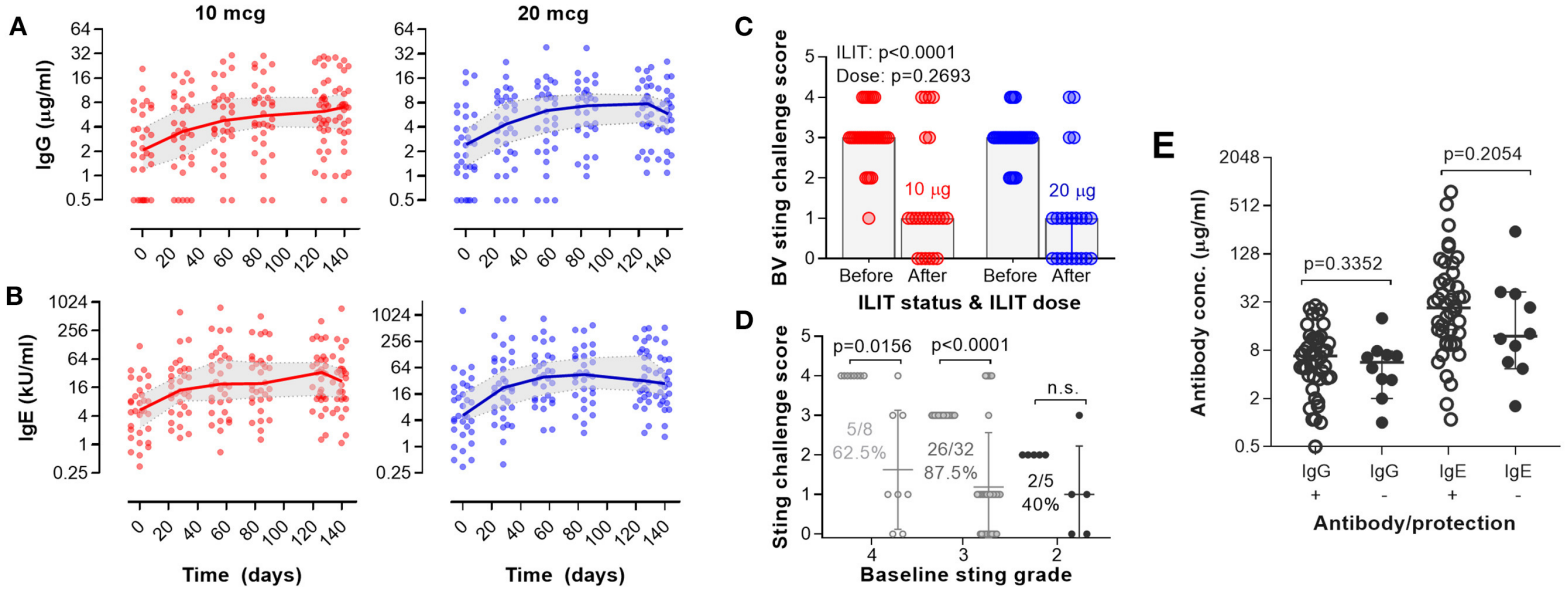
Assessment of antibody responses and honeybee sting provocation test after bee venom ILIT. Study subjects were treated with 10- (red) or 20-μg (blue) bee venom allergen extract, and allergen-specific IgG **(A)** and IgE **(B)** were analyzed by SPRIA (IgG) or ImmunoCAP (IgE). The red and blue lines show the median antibody concentration, and the gray-filled area show the 95% confidential interval of the median. **(C)** After completed ILIT, 45 study participants were subject to a honeybee sting challenge. The challenge was done at screening as well as 8 weeks after the last bee venom allergen ILIT with 10-(red) and 20-μg (blue) BeeAlleVax™. The sting reactions were scored and the effect of ILIT and dose were analyzed statistically using a mixed-effects model. **(D)** Per-protocol analysis of the combined sting challenge data as a function of sting grading at baseline. **(E)** Based on the sting reactions, patients were segregated into protected (*n* = 35) and non-protected (*n* = 10) patients, and the IgG or IgE concentrations measured in serum at the time of the challenge (day 140) were assigned to the protected or non-protected patients. The antibody levels were then compared by the Mann–Whitney U test for a statistical difference.

As shown in Figure 4A, in the 32 patients of a multicenter study who were treated with the 10-μg dose of *BeeAlleVax*™, a statistically significant increase in IgG was observed on day 28 and after a single injection (*p* < 0.001). The highest serum concentration of bee venom-specific IgG was determined on day 126, 40 days post fourth ILIT, where an average 3.4-fold increase as compared to IgG screening concentration was measured. Similar results were observed in the 33 patients receiving 20 μg of *BeeAlleVax*™. The relative increase in IgG, although not statistically significant, was slightly higher than for the 10-μg group, averaging a 4.3-fold increase on day 126 relative to screening.

In both 10- and 20-μg treatment groups, the differences of bee venom-specific IgE titers measured at the six consecutive visits were highly significant (Figure 4B; *p* < 0.001). A steady increase in IgE was observed especially for the 20-μg treatment group. The highest overall increase in IgE relative to screening was observed between day 56 and 126, averaging approximately 10-fold in the 10-μg treatment group and approximately 20-fold in the 20-μg treatment group. The highest individual increase in bee venom-specific IgE was observed in patients entering the trial with a very low serum IgE. The inclusion criterion for this study was “positive IgE” and not a quantitative measure of IgE. When excluding patients who entered the trial with a bee venom-specific IgE of CAP class 1 or 2, the relative increases in IgE are only around 5-fold in the 10-μg treatment group and around 7-fold in the 20-μg treatment group.

### Bee Sting Challenge, Randomized Study

To test the efficacy of bee venom ILIT, the study subjects were challenged in a deliberate honeybee sting provocation test. Of the 67 study subjects completing ILIT, 45 (23 in the 10-μg group and 22 in the 20-μg group) underwent a deliberate bee sting challenge. Taking the bee sting history of the subjects as a baseline, a significant reduction in grade III and IV systemic allergic reactions was observed after a deliberate bee sting (Figure 4C; *p* < 0.001; Mixed-effects model). In the 10-μg group, 21 experienced grade III (*n* = 15) or IV (*n* = 6) allergic reactions at bee stings before the study, while only 6 patients experienced grade III (*n* = 2) or IV (*n* = 4) allergic reactions after a deliberate bee sting challenge. In the 20-μg group, 19 experienced grade III (*n* = 17) or IV (*n* = 2) allergic reactions at bee stings before the study, while only 4 persons experienced grade III (*n* = 2) or IV (*n* = 2) allergic reactions after a deliberate bee sting challenge. For the combined data set, 5 out of 8 (62.5%, *p* = 0.0156), 26 out of 32 (87.5%, *p* < 0.0001), and 2 out of 5 (40%, n.s.) patients with grades IV, III, and II reactions at baseline, respectively, experienced an improvement of two or more grades upon ILIT (Figure 4D). Out of the 10 non-protected subjects, six and four were from 10- to 20-μg groups, respectively.

In total, 21 subjects of each dose group completed the bee sting challenge period with complete scoring of adverse reactions (Table 3). In total, 20 subjects in the 10-μg group and 19 in the 20-μg group experienced at least 1 AE during the bee sting challenge. Most AEs were reported as mild: 14 in the 10-μg treatment group and 15 in the 20-μg treatment group. The reported numbers of moderate and severe AEs were also similar in the 10- (*n* = 7) and 20-μg (*n* = 5) groups. AEs included allergic reaction (*n* = 8+3 in the 10- and 20-μg group), urticaria NOS (*n* = 3+6), erythema (*n* = 3+4), localized edema (*n* = 3+3), and anaphylaxis (*n* = 1+3). There was no clear pattern with regard to the type or frequency of AEs and the dose of *BeeAlleVax*™ (10 or 20 μg). However, the number of immune system AEs precipitated by the deliberate bee sting challenge indicates a lack of efficacy for *BeeAlleVax*™ ILIT in providing adequate immunological protection. For this reason, the study was terminated earlier than planned before the planned last bleeding on day 168 post first ILIT.

**Table 3:** AEs in the safety population (reported for ≥5% of subjects in either treatment groups) during the bee sting challenge period by the preferred term and after completion of ILIT.

**Preferred Term**	**10 μg *N* = 21**	**20 μg *N* = 21**
Number of Subjects with at least one AE	20 (95.2%)	19 (90.5%)
Allergy to insect sting	8 (38.1%)	3 (14.3%)
Urticaria NOS	3 (14.3%)	6 (28.6%)
Erythema	3 (14.3%)	4 (19.0%)
Localized edema	3 (14.3%)	3 (14.3%)
Peripheral swelling	2 (9.5%)	3 (14.3%)
Headache NOS	3 (14.3%)	1 (4.8%)
Nausea	3 (14.3%)	1 (4.8%)
Pruritus NOS	3 (14.3%)	1 (4.8%)
Flushing	2 (9.5%)	2 (9.5%)
Anaphylactic reaction	1 (4.8%)	3 (14.3%)
Conjunctivitis NOS	2 (9.5%)	1 (4.8%)
Dizziness	2 (9.5%)	1 (4.8%)
Face edema	1 (4.8.%)	2 (9.5%)
Throat tightness	1 (4.8%)	2 (9.5%)
Muscle twitching	2 (9.5%)	0 (0.0%)
Urticaria drug—induced	2 (9.5%)	0 (0.0%)
Abdominal pain NOS	0 (0.0%)	2 (9.5%)
Urticaria generalized	0 (0.0%)	2 (9.5%)

To test the hypothesis that protections correlate with more IgG and less IgE, IgG and IgE responses in protected (*n* = 35) and non-protected patients (*n* = 10) was analyzed for the 45 patients who underwent a deliberate honeybee sting challenge. At the time point of the challenge on day 140, a slightly higher IgG concentration was observed in the protected cohort than in the non-protected cohort (Figure 4E). However, the protected patients also had slightly higher IgE levels. Neither of these differences were statistically significant. A similar result was found when comparing the development of IgG or IgE antibody responses for protected and non-protected patients throughout the whole study.

### Further Monitoring of Clinical Parameters, Randomized Study

Clinically significant laboratory findings were observed for one study subject (10-μg treatment group) for hemoglobin, erythrocytes, and hematocrit (low levels) and one subject (20-μg treatment group) for microorganisms (urinalysis). In total, seven subjects had clinically significant high levels of tryptase. Clinically significant high levels of tryptase were recorded for four subjects in the 10-μg treatment group and one subject in the 20-μg treatment group on day 140, where mean tryptase levels were 7.6 ± 6.2 μg/L in the 10-μg treatment group and 5.3 ± 4.7 μg/L in the 20-μg treatment group, and the levels increased to 21.1 ± 28.4 μg/L and to 9.7 ± 9.4 μg/L, respectively, 120 min after the sting challenge. Other clinically significant high levels of tryptase were seen on day 28 at 120 min post-injection for one subject of the 10-μg treatment group and one subject of the 20-μg treatment group. Other hematological and chemical analysis did not suggest patterns of AEs to ILIT or differences between the two treatment groups.

## Discussion

A recent meta-analysis, including five systematic reviews, five randomized controlled trials, three controlled studies, and four case series, concluded that VIT significantly reduced the risk of severe systemic reactions to insect stings, improved the quality of life, and might be cost-effective in persons with repeated systemic reactions and impaired quality of life (24). Unfortunately, and despite these obvious benefits, a large fraction of patients with venom allergy are reluctant to undergo subcutaneous VIT, the main reasons being the time required to complete the treatment comprising around 100 injections and doctor visits over 5 years and the often associated allergic AEs, including anaphylaxis (19). The initial up-titration with these allergens requires hospitalization, which is inconvenient and costly.

While the efficacy of prophylactic vaccines typically can be improved by increased doses of antigens (25, 26), the efficacy of subcutaneous VIT, as measured by the total number of injections required to complete successful therapy, cannot be improved by solely increasing the allergen doses, for reasons of safety. More side effects are observed with higher doses of allergen, especially during the rush or ultra-rush build-up phase (27, 28). Therefore, current clinical guidelines recommend dose-adjustment schedules with lower allergen doses for patients with risk factors, e.g., mastocytosis, increased baseline serum tryptase levels, mast cell activation disorders, and patients with a history of systemic AEs to stings (2, 29). Yet, while the allergic side effects of VIT can be avoided by lowering the allergen doses, this may impair the therapeutic efficacy. In the current study, we investigated whether changing the route of administration from subcutaneous to intralymphatic administration route may offer a way to reduce the required number of injections and the allergen dose, hence, potentially reducing the number of AEs while maintaining the efficacy.

This first-in-human bee venom ILIT study showed that intralymphatic allergen delivery *per se* is a fast and safe method for bee VIT. In total, 12 patients were included in the study and received a cumulative dose of 31.1 μg in three sessions with 14-day intervals. Not a single AE or SAE was reported. Of the eight patients that agreed to test the bee venom ILIT efficacy in a bee sting challenge, seven patients showed either no or only minor systemic allergic reactions (grade I) upon the challenge. These results are comparable to the published efficacy of subcutaneous VIT, which ranges between 77 and 84% for the protection against allergic reactions in a honeybee sting challenge (2, 30, 31). We therefore decided to evaluate further the safety and efficacy of bee venom ILIT in a multicenter phase I/II clinical trial. Although the regulatory and scientific recommendation for confirmatory AIT trials is to use a double-blinded placebo-controlled design (32–34), we chose an open-label design for safety reasons as still little was known about ILIT in humans. Of note, the interval between two ILIT sessions was doubled to 28 days, and a fourth ILIT injection was added. Furthermore, the commercially available allergen extract used in the pilot study was replaced with an investigational medicinal product, specially manufactured for the trial.

The results again revealed that bee venom ILIT *per se* was well tolerated. However, SAEs were observed during the treatment period of the randomized study. These were typically not attributed to the bee venom injection, but in one instance to a non-compliant intralymphatic injection, i.e., intravascular. The subsequent bee sting challenge enables protection in 35 out of 45 patients with a reduction in grade III and IV systemic allergic reactions as compared to baseline. This protection level of 77.8% is in the lower end of the expected protection after a 5-year subcutaneous VIT (31), and the protection was only partial, because many patients still reacted with a grade I reaction. As 10 out of 45 patients were not protected and still reacted with a grade III or IV reaction to the bee sting, we concluded that ILIT with *BeeAlleVax*™ did not confer sufficient immunity.

Insufficient absorption of bee venom on the aluminum hydroxide adjuvant may be one possible explanation for a relatively high number of systemic AEs after bee venom ILIT with *BeeAlleVax*^TM^. Later analyses of the product revealed that a major fraction of the bee venom allergen remained unabsorbed to aluminum hydroxide. Of note, neither the product *BeeAlleVax* nor the venom itself was ever tested in subcutaneous VIT before its use in the current ILIT trial. Another possible explanation of a relatively high number of sting reactions after the deliberate bee sting challenge may have been the different etiologies of bees at different centers and continents. No major safety concerns were identified for the method of intranodal vaccine delivery; therefore, the contribution of the mode of delivery to the potential immune response does warrant further investigation.

Venom immunotherapy has been associated with increased IgGs (13, 35–38), and with higher doses producing higher IgG titers (39) although it is not clear if IgG levels correlate with sting protection (40–43). In both presented ILIT trials, we observed enhanced bee venom-specific IgG antibody levels but no significant difference in IgG levels in protected and in non-protected patients could be detected. While bee venom ILIT did not boost the allergen-specific IgE responses in the setting of a pilot study, a rise was observed in the later randomized study. The latter phenomenon is also frequently observed after subcutaneous VIT but with a subsequent decrease in IgE toward the end of the VIT (2, 44–46). It has been shown that the functional activity of blocking antibodies might be a more accurate measure of clinical efficacy as IgG/IgG4 levels decreased about 80–90% within 1 year after stopping AIT, while IgG-associated serum IgE-inhibitory activity persisted for several years (47). Upon conventional AIT, mainly IgG1 is initially formed, and although IgG4 also rises in the initial period, it becomes more prominent after 1–2 years of treatment and might therefore reflect long-term antigenic stimulation (48–51). As a consequence, 3–4 ILIT injections in the short period as performed in the current studies may not be sufficient to cause such changes in antibodies, and the study period is not long enough to see long-term effects on IgG4. Nonetheless, the more abundant and potentially allergen-blocking IgG1 is effective in ameliorating allergic symptoms during the bee sting challenge (47). Hence, one may assume that ILIT with bee venom requires more injections and longer treatment duration with a higher cumulative dose than pollen ILIT for a stronger immunological response of IgG. Thus, future bee venom ILIT studies should possibly also consider longer studies and more injections as to reach higher levels of IgG or better protection. Indeed, a reduction from 6,000 μg and 100 doses during 5 years to 30–80 μg and 3–4 doses during 2–3 months may be too ambitious for the initial development of hymenoptera ILIT.

As no correlation was observed between IgE antibodies and bee sting challenge reactions, this may suggest that the increased IgE levels observed in the multicenter ILIT trial had no negative impact on the efficacy of bee venom ILIT. Here, other factors should perhaps have been considered in the immune analysis. Immune tolerance with a Th2 to Th1 shift, an increase in the number of regulatory T-cells (Tregs), and an increase in the secretion of interferon gamma (INFγ), as well as a decrease in the secretion of interleukins IL-4 and IL-13 are observed during VIT (47). Moreover, IL-10 produced by Tr1-type Tregs plays an important role as IL-10 inhibits the differentiation and proliferation of IgE-secreting B cells by blocking the B7/CD28 pathway (47). The important role of increased IL-10 levels was shown in beekeepers shortly after the start of bee venom season and during VIT as the development of clinical and immunological tolerance (47). IL-10 producing Tregs can also inhibit the proliferation of PLA-specific effector T-cells, suppresses dendritic cell maturation, and MHC class II and costimulatory ligand expression (47, 48). However, perhaps the most promising surrogate measure for the sting challenge protection after VIT, as allergic reactions to venom stings are systemic and mediated by basophil cells, may be the basophil activation test (BAT). Increased protection should at least theoretically go along with reduced basophil reactivity. Although the sting provocation test is the gold standard of measuring the effectiveness of VIT, both doctors and patients are hesitant to perform such tests (2). Hence, good surrogate markers are needed. Today, we would probably include BAT and cellular allergen stimulation tests (CAST), in combination with antibody assessments such as IgE, IgG1, IgG4, and the measurement of inhibition of allergen-specific IgE binding by blocking antibodies (52) in trials analyzing the efficacy analysis of hymenoptera ILIT.

While the current two bee venom ILIT trials were the first and last to be performed in humans, several trials have been performed with grass pollen allergen ILIT. A recent systematic review and meta-analysis of 17 clinical trials about ILIT for allergic rhinitis showed a clear benefit toward symptom alleviation and reduced medication use, and comparable safety and efficacy to both SCIT and SLIT (53). For grass pollen allergen ILIT, it is recommended that the injections are given at 4-week intervals (14). Shorter intervals showed no clinical improvement despite the indications of immunological tolerance (54). The reason for this is thought to be the short interval between the injections that does not allow enough time for the development of antigen-specific immune responses. Therefore, further venom ILIT studies should also test the importance of dosing intervals on safety, immune responses, and efficacy, e.g., longer intervals to facilitate the stimulation of IgG4 antibody responses. Of note, it was recently suggested that a booster ILIT injection, 1 year after the original three grass pollen ILIT injections, prolonged the increase of allergen-specific IgG4 levels (13). Studies on subcutaneous VIT have shown that intervals can be extended up to 6 months without an increased incidence and severity of AEs (55, 56). With respect to efficacy, the duration of VIT was more important than the intervals between treatments (57). Recent observations showed less systemic reactions after the completion of VIT treatment for at least 5 years with a systemic reaction rate of 9.5% during another 5 years after discontinuing VIT (17). In contrast, after 1 year treatment with VIT, nearly 22% of the patients showed insufficient protection and allergic reactions against subsequent “field” stings during 3–4 years post-treatment (17, 58). Although the current trial shows a comparable efficacy after only 3–4 injections, one may speculate that going from 20 to 25% systemic reaction to <10% systemic reactions may be reached by prolonging the ILIT treatment. Thus, the definitive number of bee venom injections may also need to be considered in future ILIT trials. As subcutaneous VIT requires a longer treatment duration with more injections than subcutaneous pollen AIT, we assume a higher number of injections in venom ILIT than in grass pollen ILIT, the latter that all have been performed with three ILIT injections. Further injections and longer intervals will of course also increase the overall time that the immune system is exposed to the allergen, and indeed, antigen persistence has been shown to facilitate B- and T-cell responses upon vaccination (59–61).

There are several limitations to these two clinical studies. Firstly, the small sample size of a pilot study limits the significance of the results. Secondly, the allergen extract *BeeAlleVax*^*TM*^ has limited validity as it was never tested on humans before. Therefore, the information about potential safety and efficacy of the product were restricted and a differentiation between insufficient efficacy of *BeeAlleVax*^*TM*^ and complications caused by intranodal immunotherapy was difficult. There are no clear results for long-term protective efficacy due to the early termination, which leads to limited data and cancellation of the planned 2-year follow-up study after the treatment with *BeeAlleVax*^*TM*^. The allergen extract itself has limited validity as it has never tested on humans before. Despite these limitations, both studies provide useful information on bee venom ILIT as it is the first evaluation of intralymphatic allergen application in patients with bee venom allergy, a disease which is prevalent globally and can lead to the impairment of quality of life due to the fear of subsequent stings (62, 63).

In conclusion, the presented bee venom ILIT studies show the very first results of an alternative VIT route with the aim of reducing the number of injections and the overall dose of therapeutic allergen. In part, the revealed data were promising and suggest that venom ILIT may allow to lower both the dose and the number of venom injections as compared to SCIT. These venom ILIT trials were made to be representative of venom allergies on patients with bee venom allergy. However, VIT with honeybee venom causes more AEs than VIT with yellow jacket or vespid venom (20, 24, 31, 64), honeybee venom allergy is a risk factor for VIT failure (19, 65), patients with honeybee VIT show a higher relapse rate and less protection than patients with vespid VIT (15), and the prevalence of allergic reactions to wasp stings is typically higher than to honeybee stings (66, 67). Hence, future ILIT safety and efficacy studies should probably also consider vespid venom as an alternative to wasp SCIT. Moreover, as lifelong therapy should be considered in patients with severe systemic sting reactions, SAEs during VIT, a high risk of future honeybee stings, mastocytosis, or increased baseline serum tryptase levels (2), ILIT could be considered as a patiently friendly alternative to SCIT in this patient population. The major benefit of bee venom ILIT would be a lower risk for AEs and SAEs during treatment, but also that the treatment adherence may be improved when patients are not bound to the busy dosing scheme and doctor visits in conventional subcutaneous VIT. Thus, venom ILIT is most promising in making the causal treatment against IgE-mediated allergies shorter, safer, more cost-effective, and patient optimization with a potential to increase compliance. While the results demonstrate potential clinical benefits of this novel immunotherapy, it also shows that the safety and efficacy of bee venom ILIT need further evaluation and optimization. Furthermore, the quality of injections, allergen dose, injection numbers, and injection frequency need to be tested in future bee venom ILIT studies. The authors would encourage and support further clinical studies to evaluate bee venom ILIT with respect to dosing and dosing frequency as well as allergen and adjuvant selection.

## Data Availability Statement

The raw data supporting the conclusions of this article will be made available by the authors, without undue reservation.

## Ethics Statement

The studies involving human participants were reviewed and approved by Kantonale Ethikkommision, Kanton Zurich. The patients/participants provided their written informed consent to participate in this study.

## Author Contributions

AC and PJ analyzed data, wrote manuscript, and prepared figures and tables. TK planned studies, raised funding, analyzed data, and overheaded the projects. GS planned and performed the trials as a principal investigator. IE and BP performed the clinical work as study doctors. LŠ planned the project and wrote this manuscript. BW overheaded the project. All authors read, revised, and approved the final manuscript.

## Funding

The pilot study was investigator initiated. The randomized study was sponsored and approved by MannKind Corp. (Valencia, CA, USA). The sponsor had no part in clinical data collection, data quality management, and data analysis or data interpretation.

## Conflict of Interest

At the time of the study, TK was scientific advisor to MannKind Corp (Valencia, CA) and received financial support for the conduction of the described trials. TK and PJ have received research funding and travel grants from Allergy Therapeutics (Worthing, UK), which however has no involvement related to the current report. The remaining authors declare that the research was conducted in the absence of any commercial or financial relationships that could be construed as a potential conflict of interest.

## Publisher's Note

All claims expressed in this article are solely those of the authors and do not necessarily represent those of their affiliated organizations, or those of the publisher, the editors and the reviewers. Any product that may be evaluated in this article, or claim that may be made by its manufacturer, is not guaranteed or endorsed by the publisher.

## References

[1] Clark S Camargo CA. Epidemiology of anaphylaxis. Immunol Allergy Clin North Am. (2007) 27:145–63. 10.1016/j.iac.2007.03.002 17493495

[2] Sturm GJ Varga EM Roberts G Mosbech H Bilò MB Akdis CA. EAACI guidelines on allergen immunotherapy: hymenoptera venom allergy. Allergy Eur J Allergy Clin Immunol. (2018) 73:744–64. 10.1111/all.13262 28748641

[3] Schiener M Graessel A Ollert M Schmidt-Weber CB Blank S. Allergen-specific immunotherapy of Hymenoptera venom allergy–also a matter of diagnosis. Hum Vaccines Immunother. (2017) 13:2467–81. 10.1080/21645515.2017.1334745 28604163 PMC5647953

[4] Bilò MB Kamberi E Tontini C Marinangeli L Cognigni M Brianzoni MF. High adherence to hymenoptera venom subcutaneous immunotherapy over a 5-year follow-up: a real-life experience. J Allergy Clin Immunol. (2016) 4:327–9. 10.1016/j.jaip.2015.09.014 26563675

[5] Martínez-Gómez JM Johansen P Erdmann I Senti G Crameri R Kundig TM. Intralymphatic injections as a new administration route for allergen-specific immunotherapy. Int Arch Allergy Immunol. (2009) 150:59–65. 10.1159/000210381 19339803

[6] Manolova V Flace A Bauer M Schwarz K Saudan P Bachmann MF. Nanoparticles target distinct dendritic cell populations according to their size. Eur J Immunol. (2008) 38:1404–13. 10.1002/eji.200737984 18389478

[7] Hellkvist L Hjalmarsson E Kumlien Georén S Karlsson A Lundkvist K Winqvist O. Intralymphatic immunotherapy with 2 concomitant allergens, birch and grass: a randomized, double-blind, placebo-controlled trial. J Allergy Clin Immunol. (2018) 142:1338–41. 10.1016/j.jaci.2018.05.030 29908212

[8] Senti G Prinz Vavricka BM Erdmann I Diaz MI Markus R Mccormack SJ. Intralymphatic allergen administration renders specific immunotherapy faster and safer: a randomized controlled trial. Proc Nat Acad Sci. (2008) 105:17908–12. 10.1073/pnas.0803725105 19001265 PMC2582048

[9] Johansen P Von Moos S Mohanan D Kündig TM Senti G. New routes for allergen immunotherapy. Hum Vaccin Immunother. (2012) 8:1525–33. 10.4161/hv.21948 23095873 PMC3660774

[10] Hylander T Latif L Petersson-Westin U Cardell LO. Intralymphatic allergen-specific immunotherapy: an effective and safe alternative treatment route for pollen-induced allergic rhinitis. J Allergy Clin Immunol. (2013) 131:412–20. 10.1016/j.jaci.2012.10.056 23374268

[11] Skaarup SH Graumann O Schmid J Bjerrum AS Skjold T Hoffmann HJ. The number of successful injections associates with improved clinical effect in intralymphatic immunotherapy. Allergy Eur J Allergy Clin Immunol. (2021) 76:1859–61. 10.1111/all.14642 33099797

[12] Skaarup SH Schmid JM Skjold T Graumann O Hoffmann HJ. Intralymphatic immunotherapy improves grass pollen allergic rhinoconjunctivitis: a 3-year randomized placebo-controlled trial. J Allergy Clin Immunol. (2021) 147:1011–9. 10.1016/j.jaci.2020.07.002 32679209

[13] Weinfeld D Westin U Hellkvist L Mellqvist UH Jacobsson I Cardell LO. preseason booster prolongs the increase of allergen specific IgG4 levels, after basic allergen intralymphatic immunotherapy, against grass pollen seasonal allergy. Allergy, Asthma Clin Immunol. (2020) 16:1–8. 10.1186/s13223-020-00427-z 32368217 PMC7189556

[14] Hylander T Larsson O Petersson-Westin U Eriksson M Kumlien Georén S Winqvist O. Intralymphatic immunotherapy of pollen-induced rhinoconjunctivitis: a double-blind placebo-controlled trial. Respir Res. (2016) 17:1–9. 10.1186/s12931-016-0324-9 26817454 PMC4728811

[15] Lerch E Mü Ller UR. Long-term protection after stopping venom immunotherapy: results of re-stings in 200 patients. J Allergy Clin Immunol. (1998) 101:606–12. 10.1016/S0091-6749(98)70167-8 9600496

[16] Keating MU Kagey-Sobotka A Hamilton RG Yunginger JW. Clinical and immunologic follow-up of patients who stop venom immunotherapy. J Allergy Clin Immunol. (1991) 88:339–48. 10.1016/0091-6749(91)90095-6 1890261

[17] Golden DBK Kwiterovich KA Kagey-Sobotka A Valentine MD Lichtenstein LM. Discontinuing venom immunotherapy: outcome after five years. J Allergy Clin Immunol. (1996) 97:579–87. 10.1016/S0091-6749(96)70302-0 8621842

[18] Golden DBK Kwiterovich KA Kagey-Sobotka A Lichtenstein LM. Discontinuing venom immunotherapy: extended observations. J Allergy Clin Immunol. (1998) 101:298–305. 10.1016/S0091-6749(98)70239-8 9525443

[19] Kołaczek A Skorupa D Antczak-Marczak M Kuna P Kupczyk M. Safety and efficacy of venom immunotherapy: a real life study. Postep Dermatologii i Alergol. (2017) 34:159–67. 10.5114/ada.2017.67082 28507496 PMC5420609

[20] Mosbech H Muller U. Side-effects of insect venom immunotherapy: results from an EAACI multicenter study. Allergy Eur J Allergy Clin Immunol. (2000) 55:1005–10. 10.1034/j.1398-9995.2000.00587.x 11097308

[21] Senti G Crameri R Kuster D Johansen P Martinez-Gomez JM Graf N. Intralymphatic immunotherapy for cat allergy induces tolerance after only 3 injections. J Allergy Clin Immunol. (2012) 129:1290–6. 10.1016/j.jaci.2012.02.026 22464647

[22] Kündig TM Johansen P Bachmann MF Cardell LO Senti G. Intralymphatic immunotherapy: Time interval between injections is essential. J Allergy Clin Immunol. (2014) 133:930. 10.1016/j.jaci.2013.11.036 24439076

[23] Siegrist CA. Vaccine immunology. In: Plotkin SA, editor. Vaccines (6th ed). (2013) p. 14–32. 10.1016/B978-1-4557-0090-5.00004-5

[24] Dhami S Zaman H Varga EM Sturm GJ Muraro A Akdis CA. Allergen immunotherapy for insect venom allergy: a systematic review and meta-analysis. J Allergy Clin Immunol. (2017) 72:342–65. 10.1111/all.13077 28120424

[25] Foppa IM Franks R Pratt D Forshee RA. Comparative effectiveness of high-dose versus standard-dose influenza vaccines in US residents aged 65 years and older from 2012 to 2013 using Medicare data: a retrospective cohort analysis. Lancet Infect Dis. (2016) 15:293–300. 10.1016/S1473-3099(14)71087-4 25672568 PMC4834448

[26] Choi JH Choo EJ Huh A Choi SM Eom JS Lee JS. Immunogenicity and safety of diphtheria-tetanus vaccine in adults. J Korean Med Sci. (2010) 25:1727–32. 10.3346/jkms.2010.25.12.1727 21165286 PMC2995225

[27] Stock R Fischer T Aβmus K Zoeller N Ackermann H Kaufmann R. Safety and tolerability of venom immunotherapy: evaluation of 581 rush- and ultra-rush induction protocols (safety of rush and ultra-rush venom immunotherapy). World Allergy Organ J. (2021) 14:1–13. 10.1016/j.waojou.2020.100496 33376576 PMC7750415

[28] Mellerup MT Hahn GW Poulsen LK Malling HJ. Safety of allergen-specific immunotherapy. Relation between dosage regimen, allergen extract, disease and systemic side-effects during induction treatment. Clin Exp Allergy. (2000) 30:1423–9. 10.1046/j.1365-2222.2000.00910.x 10998019

[29] Selcuk A Baysan A. Venom immunotherapy in indolent systemic mastocytosis with high serum tryptase level. Hum Vaccines Immunother. (2021) 17:1599–603. 10.1080/21645515.2020.1846398 33651660 PMC8115573

[30] Müller U Helbling A. Berchtold E. Immunotherapy with honeybee venom and yellow jacket venom is different regarding efficacy and safety. J Allergy Clin Immunol. (1992) 89:529–35. 10.1016/0091-6749(92)90319-W 1740583

[31] Ruëff F Vos B Oude Elberink J Bender A Chatelain R Dugas-Breit S. Predictors of clinical effectiveness of Hymenoptera venom immunotherapy. Clin Exp Allergy. (2014) 44:736–46. 10.1111/cea.12275 24447114

[32] European Medicines Agency. Guideline on the Clinical Development of Products for Specific Immunotherapy for the Treatment of Allergic Diseases. London, England. (2008).

[33] Kaul S May S Lüttkopf D Vieths S. Regulatory environment for allergen-specific immunotherapy. Allergy Eur J Allergy Clin Immunol. (2011) 66:753–64. 10.1111/j.1398-9995.2011.02552.x 21288251

[34] Pfaar O Agache I Bergmann KC Bindslev-Jensen C Bousquet J Creticos PS. Placebo effects in allergen immunotherapy—An EAACI Task Force Position Paper. Allergy Eur J Allergy Clin Immunol. (2021) 76:629–47. 10.1111/all.14331 32324902

[35] Demšar Luzar A Korošec P Košnik M Zidarn M Rijavec M. Hymenoptera venom immunotherapy: immune mechanisms of induced protection and tolerance. Cells. (2021) 10. 10.3390/cells10071575 34206562 PMC8306808

[36] Feng M Zeng X Su Q Shi X Xian M Qin R. Allergen immunotherapy–induced immunoglobulin G4 reduces basophil activation in house dust mite–allergic asthma patients. Front Cell Dev Biol. (2020) 8:1–9. 10.3389/fcell.2020.00030 32154245 PMC7044416

[37] P W Ewan J Deighton A B Wilson PJL. Venom-specific IgG antibodies in bee and wasp allergy: lack of correlation with protection from stings. Clin Exp Allergy. (1993) 23:647–60. 10.1111/j.1365-2222.1993.tb01791.x 8221268

[38] Varga EM Francis JN Zach MS Klunker S Aberer W Durham SR. Time course of serum inhibitory activity for facilitated allergen-IgE binding during bee venom immunotherapy in children. Clin Exp Allergy. (2009) 39:1353–7. 10.1111/j.1365-2222.2009.03303.x 19538349

[39] Golden DBK Kagey-Sobotka A Valentine MD Lichtenstein LM. Dose dependence of Hymenoptera venom immunotherapy. J Allergy Clin Immunol. (1981) 67:370–4. 10.1016/0091-6749(81)90082-8 7229226

[40] Pitsios C. Allergen immunotherapy: biomarkers and clinical outcome measures. J Asthma Allergy. (2021) 14:141–8. 10.2147/JAA.S267522 33633455 PMC7901403

[41] Gehlhar K Schlaak M Becker WM Bufe A. Monitoring allergen immunotherapy of pollen-allergic patients: the ratio of allergen-specific IgG4 to IgG1 correlates with clinical outcome. Clin Exp Allergy. (1999) 29:497–506. 10.1046/j.1365-2222.1999.00525.x 10202364

[42] Bodtger U Ejrnaes AM Hummelshoj L Jacobi HH Poulsen LK Svenson M. Is immunotherapy-induced birch-pollen-specific IgG4 a marker for decreased allergen-specific sensitivity? Int Arch Allergy Immunol. (2005) 136:340–6. 10.1159/000084227 15741732

[43] Djurup R Malling H. -J. High IgG4 antibody level is associated with failure of immunotherapy with inhalant allergens. Clin Exp Allergy. (1987) 17:459–68. 10.1111/j.1365-2222.1987.tb02040.x 3677372

[44] Michils A Baldassarre S Ledent C Mairesse M Gossart B Duchateau J. Early effect of ultrarush venom immunotherapy on the IgG antibody response. Allergy Eur J Allergy Clin Immunol. (2000) 55:455–62. 10.1034/j.1398-9995.2000.00412.x 10843426

[45] Muller U Helbling A BM. Predictive value of venom-specific IgE, IgG and IgG subclass antibodies in patients on immunotherapy with honey bee venom. Allergy Eur J Allergy Clin Immunol. (1989) 44:412–8. 10.1111/j.1398-9995.1989.tb04172.x 2802114

[46] Kemeny DM Lessof MH Patel S Youlten LJ Williams A LE. IgG and IgE antibodies after immunotherapy with bee and wasp venom. Int Arch Allergy Appl Immunol. (1989) 88:247–9. 10.1159/000234799 2707888

[47] Sahiner UM Durham SR. Hymenoptera venom allergy: how does venom immunotherapy prevent anaphylaxis from bee and wasp stings? Front Immunol. (2019) 10:1–11. 10.3389/fimmu.2019.01959 31497015 PMC6712168

[48] Jutel M Jaeger L Suck R Meyer H Fiebig H Cromwell O. Allergen-specific immunotherapy with recombinant grass pollen allergens. J Allergy Clin Immunol. (2005) 116:608–13. 10.1016/j.jaci.2005.06.004 16159631

[49] Huber S Lang R Steiner M Aglas L Ferreira F Wallner M. Does clinical outcome of birch pollen immunotherapy relate to induction of blocking antibodies preventing IgE from allergen binding? A pilot study monitoring responses during first year of AIT. Clin Transl Allergy. (2018) 8:1–15. 10.1186/s13601-018-0226-7 30338052 PMC6174570

[50] Van de Veen W Akdis M. Tolerance mechanisms of allergen immunotherapy. Allergy Eur J Allergy Clin Immunol. (2020) 75:1017–8. 10.1111/all.14126 31758812

[51] Aalberse ROBC. Serologic aspects of IgG4 antibodies. I. Prolonged immunization results in an IgG4-restricted response. J Immunol. (1983) 130:722–6. 6600252

[52] Shamji MH Francis JN Würtzen PA Lund K Durham SR Till SJ. Cell-free detection of allergen-IgE cross-linking with immobilized phase CD23: inhibition by blocking antibody responses after immunotherapy. J Allergy Clin Immunol. (2013) 132:1003–5. 10.1016/j.jaci.2013.05.025 23827773

[53] Werner MT Bosso J V. Intralymphatic immunotherapy for allergic rhinitis: A systematic review and meta-analysis. Allergy Asthma Proc. (2021) 42:283–92. 10.2500/aap.2021.42.210028 34187620

[54] Witten M Malling HJ Blom L Poulsen BC Poulsen LK. Is intralymphatic immunotherapy ready for clinical use in patients with grass pollen allergy? J Allergy Clin Immunol. (2013) 132:1248–52. 10.1016/j.jaci.2013.07.033 24035151

[55] Baenkler HW Meußer-Storm S Eger G. Continuous immunotherapy for hymenoptera venom allergy using six month intervals. Allergol Immunopathol (Madr). (2005) 33:7–14. 10.1157/13070602 15777517

[56] Kontou-Fili K Pitsios C Kompoti E Giannakopoulos Di Kouridakis S. Safety and efficacy of a progressively prolonged maintenance interval of venom immunotherapy. Int Arch Allergy Immunol. (2018) 176:39–43. 10.1159/000488143 29649812

[57] Lifelong C. CME exam: when can immunotherapy of insect sting allergy be stopped? J Allergy Clin Immunol Pract. (2015) 3:329–30. 10.1016/j.jaip.2015.02.006 25956311

[58] Golden DBK Johnson K Addison BI Valentine MD Kagey-Sobotka A Lichtenstein LM. Clinical and immunologic observations in patients who stop venom immunotherapy. J Allergy Clin Immunol. (1986) 77:435–42. 10.1016/0091-6749(86)90177-6 3950251

[59] Budroni S Buricchi F Cavallone A Bourguignon P Caubet M Dewar V. Antibody avidity, persistence, and response to antigen recall: comparison of vaccine adjuvants. NPJ Vaccines. (2021) 6:1–11. 10.1038/s41541-021-00337-0 34021167 PMC8140094

[60] Zinkernagel RM. On immunological memory. Annu Rev Immunol. (1996) 14:333–67. 10.1146/annurev.immunol.14.1.333 8717518

[61] Johansen P Storni T Rettig L Qiu Z Der-Sarkissian A Smith KA. Antigen kinetics determines immune reactivity. Proc Nat Acad Sci. (2008) 105:5189–94. 10.1073/pnas.0706296105 18362362 PMC2278203

[62] Pucca MB Cerni FA Oliveira IS Jenkins TP Argemí L Sørensen C V. Bee updated: current knowledge on bee venom and bee envenoming therapy. Front Immunol. (2019) 10:1–15. 10.3389/fimmu.2019.02090 31552038 PMC6743376

[63] Hockenhull J Elremeli M Cherry MG Mahon J Lai M Darroch J. A systematic review of the clinical effectiveness and costeffectiveness of Pharmalgen® for the treatment of bee and wasp venom allergy vol 16. Health Technology Assessment NIHR Journals Library. (2012). 10.3310/hta16120 22409877 PMC4781549

[64] Birnbaum J Ramadour M Magnan A Vervloet D. Hymenoptera ultra-rush venom immunotherapy (210 min): a safety study and risk factors. Clin Exp Allergy. (2003) 33:58–64. 10.1046/j.1365-2222.2003.01564.x 12534550

[65] Nittner-Marszalska M Cichocka-Jarosz E Małaczyńska T Kraluk B Rosiek-Biegus M Kosińska M. Safety of ultrarush venom immunotherapy: Comparison between children and adults. J Investig Allergol Clin Immunol. (2016) 26:40–7. 10.18176/jiaci.0006 27012015

[66] Bilò BM Bonifazi F. Epidemiology of insect-venom anaphylaxis. Curr Opin Allergy Clin Immunol. (2008) 8:330–7. 10.1097/ACI.0b013e32830638c5 18596590

[67] Fernandez J Blanca M Soriano V Sanchez J Juarez C. Epidemiological study of the prevalence of allergic reactions to Hymenoptera in a rural population in the Mediterranean area. Clin Exp allergy J Br Soc Allergy Clin Immunol. (1999) 29:1069–74. 10.1046/j.1365-2222.1999.00614.x 10457110

